# Oocyte shuttle, a recombinant protein transporting donor DNA into the *Xenopus* oocyte *in situ*

**DOI:** 10.1242/bio.022376

**Published:** 2017-02-08

**Authors:** Duri Rungger, Lisbeth Muster, Oleg Georgiev, Elisabeth Rungger-Brändle

**Affiliations:** 1 Station de Zoologie expérimentale, Department of Genetics and Evolution, University of Geneva, 154 route de Malagnou, Chêne-Bougeries 1224, Switzerland; 2 Institute of Molecular Life Sciences, University of Zurich-Irchel, Winterthurer Strasse 190, Zurich 8057, Switzerland; 3 Biologie cellulaire, University Eye Clinic, 20 rue Alcide Jentzer, Geneva 4 1211, Switzerland

**Keywords:** Gene delivery, Homologous recombination, Oogenesis, Vitellogenin pathway

## Abstract

The newly developed oocyte shuttle protein contains a streptavidin moiety that tightly binds biotinylated DNA. Injected intravenously into adult *Xenopus* females, the protein-DNA complex is rapidly transported through the bloodstream and, within the ovary, the vitellogenin ligand present in the protein binds to the receptors at the surface of the oocytes. The bound complex is internalized and translocates into the oocyte nucleus thanks to an SV40 nuclear localization signal, enhanced by an adjacent casein kinase phosphorylation site. Functioning of the shuttle protein is documented by transporting DNA molecules that, upon intramolecular homologous recombination within the oocyte nucleus, express easily traceable markers such as green fluorescence or tetracycline resistance.

## Abstract (precis)

**Summary:** A newly developed oocyte shuttle protein, binding donor DNA and carrying it from the bloodstream to the oocyte nucleus within the ovary, should greatly facilitate production of transgenic *Xenopus* embryos.

## INTRODUCTION

In an earlier study (Hagmann et al., 1996), we tested the frequency of homologous recombination versus illegitimate end-to-end ligation using a linearized donor plasmid Reco-σ, which may undergo either end-to-end ligation yielding a plasmid conferring kanamycin resistance, or intramolecular homologous recombination producing a plasmid coding for tetracycline resistance. In the oocyte nucleus, homologous recombination events were by far more frequent (>98%) than end-to-end ligation (<2%). In the fertilized egg and cleaving blastomeres, however, only end-to-end ligation occurred. Homologous recombination of extra-chromosomal DNA in *Xenopus* oocytes was also described by Carroll (1996) and was shown to be enhanced through targeted cleavage by chimeric nucleases (Bibikova et al., 2001).

*In vitro* assays by the technique described by Bertrand et al. (1995) have shown that recombinase activity is present only in the oocyte and disappears upon maturation (D.R. and A.T. Akhmedov, Basel Institute of Immunology, unpublished observations). To obtain efficient homologous recombination, the donor DNA should thus be introduced into oocytes.

The various techniques used to produce transgenic *Xenopus* individuals involve transplantation into unfertilized eggs of nuclei of embryonic cells (Kroll and Amaya, 1999; Hirsch et al., 2002) or sperm swelled in donor DNA solution (Amaya and Kroll, 1999; Sparrow et al., 2000; Smith et al., 2000). Mosaic transgenics including germ line were obtained by injecting I-*Sce*I meganuclease and donor DNA into fertilized eggs (Pan et al., 2006; Ogino et al., 2006). More recently, targeted gene disruption in *Xenopus* was achieved by injecting zinc-finger nuclease (ZFN) mRNAs and donor DNA into cleaving embryos (Young and Harland, 2012). TALENs were used as well to nick the target gene (Lei et al., 2012; Nakajima and Yaoita, 2015). Moreover, the CRISPR/Cas9 technique has been applied to *Xenopus* (Blitz et al., 2013; Nakayama et al., 2013).

Injection of a large number of oocytes is time-consuming and producing embryos from isolated oocytes involves either *in vitro* maturation followed by injection of sperm or transfer through the female. These steps yield relatively few embryos. Moreover, injection of donor DNA and agents cleaving the target gene yield mosaic transgenic embryos. The scope of the present study was thus to develop an oocyte shuttle protein (OS) that would transport donor DNA from the bloodstream into the oocyte nucleus within the ovary *in situ*, reaching a large number of oocytes in which homologous recombination takes place efficiently. The most promising approach to achieve this goal was to take advantage of the vitellogenin pathway. Vitellogenin is massively synthesized by the liver and transported through the bloodstream to the ovary (reviewed by Tata, 1985), where it is eventually absorbed by the oocyte through receptor-mediated endocytosis (Opresko and Wiley, 1987a,b).

**Fig. 1.**
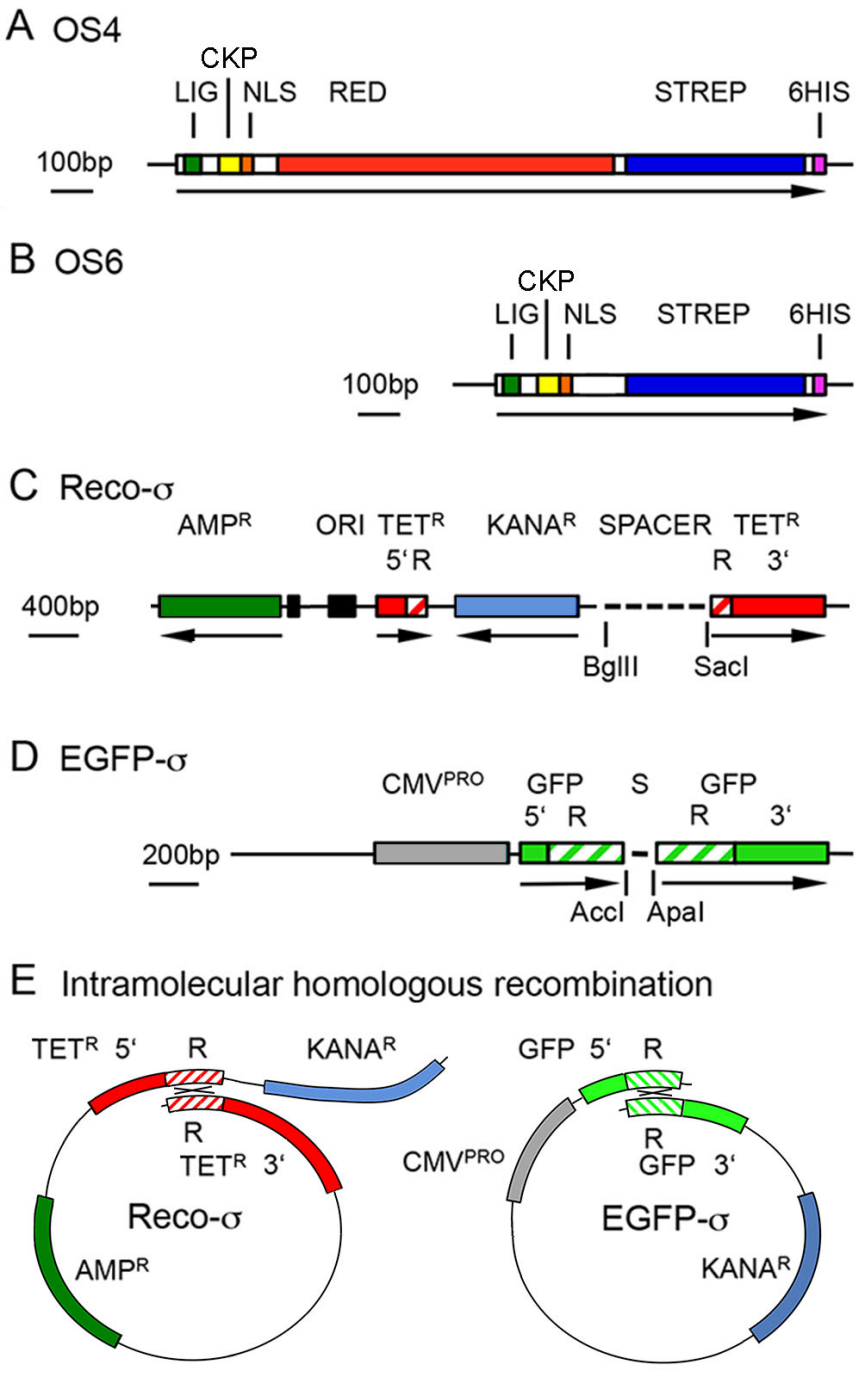
**Recombinant** **plasmids.** (A,B) Constructs encoding the shuttle proteins OS4 (A) and OS6 (B). LIG, vitellogenin ligand; CKP, casein kinase phosphorylation site; NLS, SV40 nuclear localization signal; RED, red fluorescent protein (in OS4 only); STREP, streptavidin core; 6HIS, His tag. The different segments were either cut from corresponding plasmids, synthetic oligonucleotides, or synthesized by PCR, adding appropriate linkers. For sequences see Fig. S1 (OS4) and Fig. S2 (OS6). (C) Donor plasmid Reco-σ with unique 5′ segment (5′), internal repeat (R) and unique 3′ segment (3′). Amp^R^ and Kana^R^, genes coding for ampicillin and kanamycin resistance respectively. (D) Plasmid pEGFP-σ contains two 320 bp repeats of the coding segment of pEGFP-C1 between the *Bcl*I and *Gsu*I sites, separated by a linker (S) with *Acc*I and *Ap*aI restriction sites. 5′, R, 3′, as in C. (E) Intramolecular homologous recombination within linearized Reco-σ and EGFP-σ DNAs. Crossing-over (×) takes place between the homologous repeats (R).

To mimic this pathway, the oocyte shuttle proteins (Fig. 1A,B) carry the *Xenopus* vitellogenin ligand that binds to oocyte receptors (Li et al., 2003). Nuclear translocation within the oocyte is mediated by the nuclear localization signal of SV40 (Kalderon et al., 1984), including the adjacent casein kinase phosphorylation signal. Phosphorylation of the nuclear localization signal greatly enhances its activity (Rihs et al., 1991; Xiao et al., 1997). In addition, the shuttle protein needs to tightly bind the donor DNA to be transported. To this end, we chose the minimal core of streptavidin (Sano et al., 1995) that binds biotinylated DNA. The oocyte shuttle protein, version OS4 (Fig. 1A), carried a segment coding for red fluorescent protein to be able to follow its intracellular localization. This protein was, however, poorly soluble and, in a new version of the vector, OS6 (Fig. 1B), the DsRed segment was replaced by a linker. A His-tag was added for purification of the proteins produced by baculoviral expression.

## RESULTS AND DISCUSSION

### Transport of DNA from oocyte cytoplasm to nucleus

**Fig. 2.**
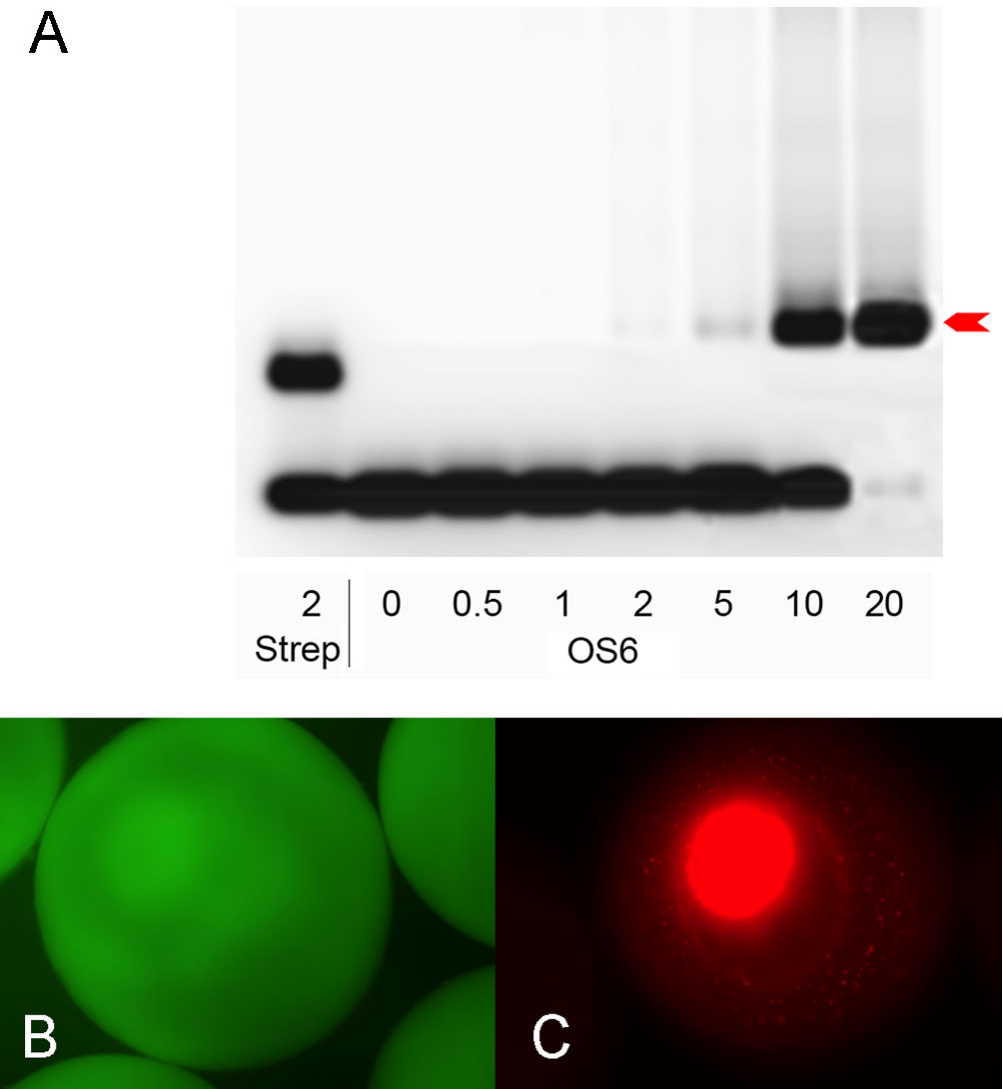
**DNA binding and nuclear translocation.** (A) Gel retardation by commercial streptavidin and OS6 protein (arrow) of an oligonucleotide that was biotinylated at the 5′ end of one strand and carried the fluorescent dye Cy5 on the 5′ end of the other. The numbers on the abscissa correspond to nominal excess of streptavidin or OS6 molecules if the extracts were 100% pure. From this titration the amount needed to bind a given number of donor DNA molecules was calculated. (B) After injection of pEGFP-C1 into *albino* oocytes, the green fluorescence expressed from this plasmid remains in the cytoplasm. (C) The red fluorescent shuttle protein, produced from a plasmid expressing OS4 under the control of a CMV promoter, migrates to the oocyte nucleus.

To document the binding of biotinylated DNA by the core streptavidin (Sano et al., 1995) present in both shuttle proteins, gel retardation assays were performed using a synthetic oligonucleotide carrying a biotin at the 5′ end of one strand, and the fluorescent dye Cy5 on the other. The poorly soluble protein OS4 yielded only a faint band-shift (not shown), but the highly soluble and thus more concentrated OS6 protein gave strong signals (Fig. 2A) allowing us to titrate the amount of protein needed to bind a given amount of DNA. According to these data, functional shuttle protein amounts to about 5% of our total protein preparation. Binding of the biotinylated oligonucleotide is very tight and resisted to 0.5% SDS (not shown).

Nuclear translocation of the shuttle protein was ascertained by injecting 2 ng (in 10 nl of injection buffer) pEGFP-C1 or a plasmid coding for OS4 under the control of a CMV promoter into the oocyte nucleus. EGFP, expressed from the injected plasmid, remained in the cytoplasm (Fig. 2B), whereas the red fluorescent OS4 protein accumulated in the oocyte nucleus (Fig. 2C). The nuclear localization signal thus functions efficiently.

The capacity of the vector protein to transport bound DNA from the cytoplasm to the nucleus was first tested by oocyte injection experiments. We used two plasmids, Reco-σ (Fig. 1C) and EGFP-σ (Fig. 1D) that express a functional, easily traceable protein only after having undergone intramolecular homologous recombination.

Reco-σ (Hagmann et al., 1996) (Fig. 1C) contains a gene conferring ampicillin resistance (Amp^R^) and two separated segments of the tetracycline resistance (Tet^R^) coding sequences, sharing a 276 bp long internal duplication. The two gene segments were separated by a gene conferring kanamycin resistance and by a 1.1 kb long spacer. Excising the spacer yields a linear donor fragment that may re-circularize by illegitimate end-to-end joining, in which case the gene conferring kanamycin-resistance is conserved but the two segments of the Tet^R^ gene remain separated. By contrast, intramolecular homologous recombination (Fig. 1E) within the repeats yields a functional gene coding for tetracycline resistance, whereas kanamycin resistance is lost. In both cases, the gene conferring ampicillin resistance (Amp^R^) is conserved.

The donor fragment with its *Bgl*II (5′-protruder) and *Sac*I (3′-protruder) ends was biotinylated at one end by Klenow reaction. We chose to insert a single biotinylated nucleotide per donor DNA molecule, reasoning that molecules carrying several biotins would more rapidly saturate the vitellogenin receptors available on the oocyte surface. Moreover, long molecules carrying several biotins might bind to several receptors and hamper endocytosis. For these reasons, we did not test molecules carrying more than one biotin.

**Fig. 3.**
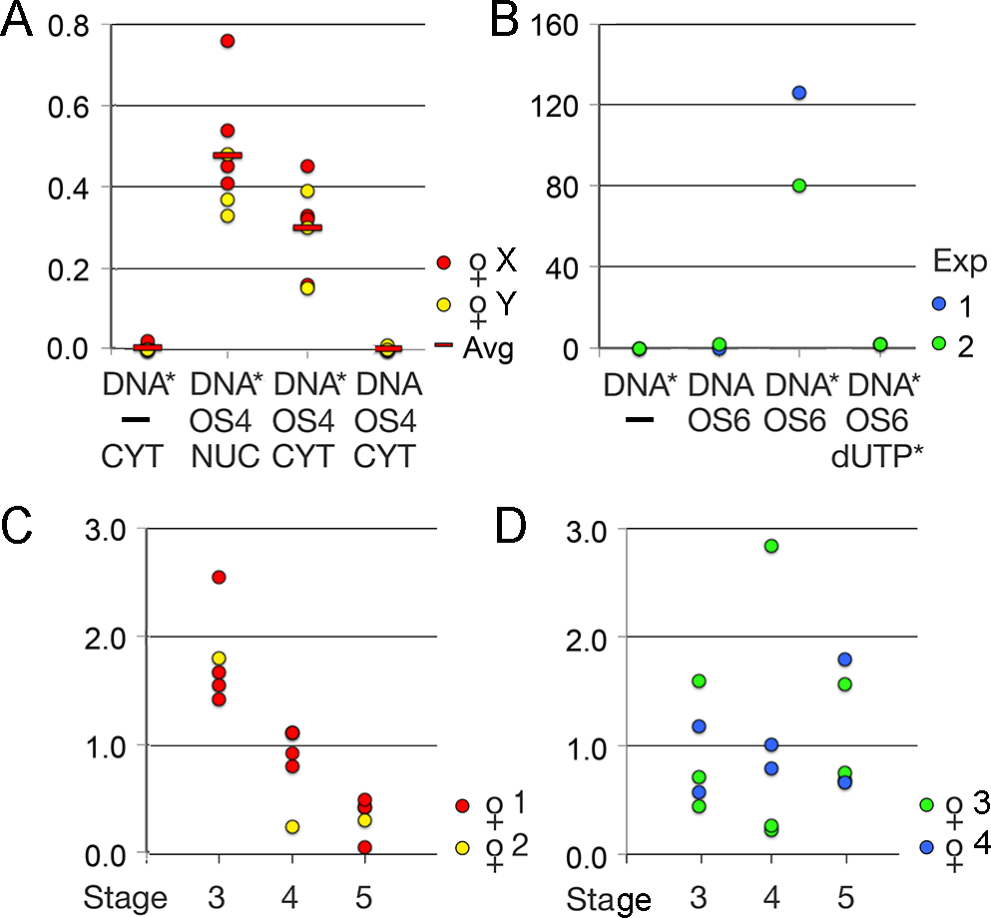
**Transport of biotinylated DNA.** (A) Transport from cytoplasm to nucleus in oocytes collected from two females (X,Y). Linearized Reco-σ, biotinylated (DNA*) or untreated (DNA), was injected without (–) or with OS4 shuttle protein into the cytoplasm (CYT) or nucleus (NUC). The numbers of Tet^R^ colonies obtained with DNA recovered from these oocytes were normalized (ratio) to the number of colonies obtained in the same series by direct nuclear injection of linearized Reco-σ. Red bars (Avg) depict mean values in each experimental point. (B) Transport from blood to oocyte nucleus *in situ*. Two independent competition experiments in which biotinylated Reco-σ alone (DNA*), non-biotinylated Reco-σ (DNA), or biotinylated Reco-σ mixed with OS6 protein, or a mixture of OS6 protein pre-incubated with excess ^14biotin^dUTP was injected, each mix into two females. Values indicated are Tet^R^ colonies per plate. (C,D) Biotinylated Reco-σ (DNA*) linked to OS6 protein was injected into four females. After two days, DNA was extracted from batches of stage 3, 4 or 5 oocytes and used to transform bacteria that were first grown on ampicillin and then replica-plated on tetracycline plates. The uptake of donor DNA was highest in stage 3 oocytes of females 1 and 2 (C), whereas in females 3 and 4 (D), the oocytes of all stages took up variable amounts of DNA with no clear preference for a given stage. The values are given as the ratio of Tet^R^ colonies per batch over the average number of TetR colonies in all batches of the same female.

The biotinylated donor was incubated with OS4 shuttle protein, and 10 nl of this mixture (0.5 ng DNA/0.1 ng OS4) were microinjected into either the oocyte nucleus or cytoplasm. After 18 h, DNA was recovered from batches of 20 oocytes and aliquots used to transform bacteria. Amp^R^ colonies were replica-plated on tetracycline plates and growing colonies were counted. The outcome of such oocyte injection experiments is summarized in Fig. 3A. Circular pReco-σ injected into the oocyte nucleus yields numerous Amp^R^ colonies but, after replica printing, no Tet^R^ colonies were found. By contrast, the linearized donor plasmid injected into the nucleus efficiently underwent homologous recombination. Neither linearized Reco-σ injected alone into the cytoplasm nor non-biotinylated donor mixed with shuttle protein produced any Tet^R^ colonies, meaning that the DNA did not reach the nucleus, where recombination would occur. However, when bound to OS4, the linear donor was efficiently transported from the cytoplasm to the nucleus and underwent intramolecular homologous recombination yielding numerous Tet^R^ colonies; only a third less than obtained by direct nuclear injection of the same amount of DNA. The normalization of the results, given as ratio of colonies obtained in each experimental point over the colonies obtained by direct nuclear injection of linearized Reco-σ, eliminates daily variation in extraction yield and transfection efficiency. Nevertheless, the results between different experimental series vary, depending most probably on the physiological state of the females.

In these experiments, 40-80% of the replica-plated Amp^R^ colonies grew on tetracycline medium. Plasmids conferring resistance to ampicillin but not to tetracycline must have arisen by end-to-end ligation. In earlier experiments (Hagmann et al., 1996), only about 20% of injected linear DNA molecules circularized in this way, whereas 80% underwent homologous recombination in the short repeats. The lowered Tet^R^/Amp^R^ ratio observed here may be due to the presence of the bound OS protein, different amounts of DNA present in the two experimental setups and loss during replica printing.

### Transport of DNA from bloodstream to the oocyte nucleus *in vivo*

The most crucial task of the shuttle protein is homing the ovary and oocyte nuclei when injected into the bloodstream of *Xenopus* females. Due to the tough skin of adult *Xenopus*, intravascular injection into the large femoral vein is hardly feasible. However, the skin on the toes can be penetrated more easily and toe veins are just large enough to be injected with a 29G insulin syringe. Slow injection of 50 µl of physiological solution leads to minimal leakage. Injection success was first verified by injecting Evans Blue, which appeared in the capillary network of the retina after a few minutes (not shown), demonstrating rapid distribution throughout the body.

An important biological aspect was to decide how the recipient *Xenopus laevis* females should be hormone treated. Internalization of vitellogenin receptors is enhanced by human chorionic gonadotropin (HCG) and insulin (Opresko and Wiley, 1987a,b) but HCG also induces ovulation. This may be of advantage because vitellogenin uptake is most likely reduced in fully-grown oocytes. Inducing ovulation would thus eliminate oocytes, which would take up the protein-DNA complex less avidly. On the other hand, hormone treatment induces vitellogenin synthesis in the liver and the massive amount of vitellogenin transported through the bloodstream might out-compete the uptake of the shuttle protein. Considering such possible drawbacks, we decided to inject a low dose (50IU) of HCG 4 h before injecting the protein-DNA complex and to induce ovulation by the high 250IUdose 10 min later. In this way, ligand uptake should be stimulated by the first dose of HCG but vitellogenin levels in the blood should increase only after internalization of the protein-DNA complex.

Following this protocol, two independent experimental series were carried out in which either biotinylated Reco-σ alone, non-biotinylated or biotinylated Reco-σ mixed with OS6 protein, or a mixture of OS6 protein pre-incubated with excess biotinylated dUTP and the biotinylated donor added last, were injected into the toe vein. The females were euthanized 2 days later. DNA recovered from batches of oocytes was used to transform bacteria that were grown first on ampicillin and then replica printed on tetracycline plates. The results (Fig. 3B) show that only biotinylated donor bound to OS protein is transported to the oocyte nucleus in the ovary *in situ*. Transport is inhibited when binding of the donor to the vector is out-competed by biotinylated dUTP.

To determine which stages of oocytes take up the donor-shuttle protein complex most avidly, biotinylated Reco-σ (500 ng) was mixed with a twofold nominal excess of OS6 protein and the complex injected into the toe vein of hormone treated females that were euthanized after 48 h. Ovaries were digested by collagenase and batches of stage 3, 4 or 5 oocytes were isolated and analyzed as in the previous experiment. Note that in hormone-treated females, fully-grown stage 6 oocytes are spawned and were thus not tested. The results obtained with oocytes obtained from four females are shown in Fig. 3C,D. The efficacy with which oocytes of different stages take up the ligand and the bound DNA seems mainly to depend on the physiological state of the female. In two females, stage 3 oocytes took up the donor most avidly (Fig. 3C) whereas, in the other two, there was no clear-cut difference between the different oocyte stages (Fig. 3D). Receptor function thus seems to depend not only on the physiological state of the recipient female but may also vary depending on ovary region and individual oocytes. Yet, donor DNA bound to the shuttle protein was again efficiently transported through the bloodstream to the ovary and into the oocyte nucleus where recombination of the test plasmids took place. By contrast, OS6-bound Reco-σ, injected into the dorsal lymph sac of another female, yielded no recombined plasmids at all. Either the DNA-shuttle protein complex was not transported to the ovary or readily degraded within the lymph.

**Fig. 4.**
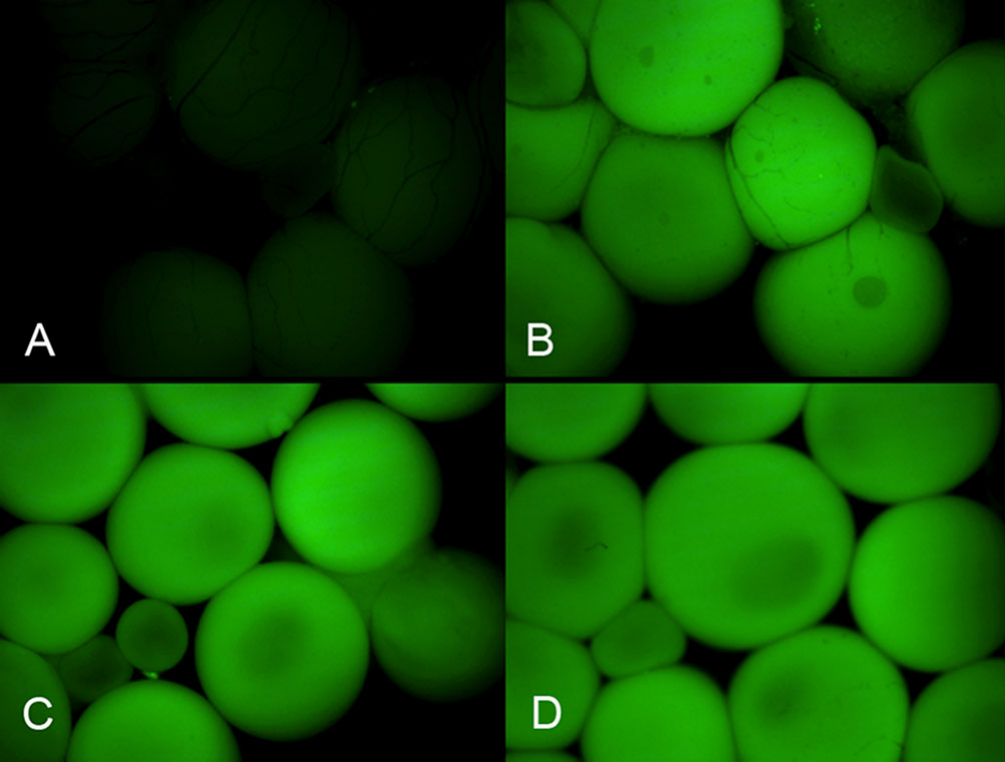
**Expression of transported EGFP-σ in ovary.** Fluorescent micrographs. (A) Control ovary recovered from a female injected two weeks earlier with biotinylated EGFP-σ without shuttle protein into the bloodstream. (B) Ovary and (C,D) isolated oocytes from three different females injected with biotinylated EGFP-σ bound to OS6 protein. Exposition and image treatment of the four shots were equal.

To visualize the distribution of DNA uptake within the target ovary, we constructed EGFP-σ (Fig. 1D) that contains a 306 bp long duplication within the EGFP-C1 gene, separated by a 60 bp long spacer. This plasmid, when injected into oocytes, does not encode functional EGFP. For shuttle experiments it was linearized by cutting the spacer with *Apa*I (blunt) and *Acc*I (sticky). A single biotinylated dUTP was inserted into the sticky end (*Acc*I) of the molecule by Klenow reaction. The biotinylated EGFP-σ, bound to OS6, was injected into three hormone-treated females. As a control, a fourth female was injected with biotinylated donor without shuttle protein. After injection, the females were kept for 2 weeks to allow for synthesis and folding of GFP protein. As shown in Fig. 4, the ovary of the female injected with biotinylated donor alone did not exhibit green fluorescence (Fig. 4A), whereas in the females injected with the protein-donor complex, virtually all oocytes within the ovary and all stages of oocytes expressed green fluorescence (Fig. 4B-D).

The transport of biotinylated DNA to the oocyte *in situ* is massive. From our earlier study (Hagmann et al., 1996), we calculated that only about one out of four thousand injected molecules undergoes intramolecular homologous recombination. In the present experiments, we obtained between 10 and 200 Tet^R^ colonies per oocyte analyzed. This means that thousands of donor molecules must have entered each oocyte. There seems to be little or no degradation during transport through the bloodstream, as the repeated segments needed for crossing over were located near the ends of the molecule and obviously remained intact.

Conclusively, the oocyte shuttle protein carries out all the expected functions. It tightly binds DNA and, when injected into the bloodstream of living *Xenopus* females, homes the ovary, mimicking the vitellogenin pathway. The complex bound to the vitellogenin receptors at the surface of the oocytes is most probably endocytosed and must have escaped intact from the endosome since it reached the nucleus, the only cellular compartment in which homologous recombination may occur.

### Outlook

Easy transport of DNA into a large number of oocytes may be of interest for different applications. Over- or misexpression of exogenous proteins could be used to test their influence on immediate post-fertilization mechanisms, or to produce egg extracts for *in vitro* studies. However, transcription takes place on circular templates only whereas biotinylation is most easily done by Klenow reaction on linearized plasmids. Circularization of linear DNA by end-to-end ligation is less efficient in the oocyte than intramolecular homologous recombination (Hagmann et al., 1996). Accordingly, to enhance expression of heterologous proteins, one might biotinylate circular DNA or circularize linear biotinylated molecules before transport. Another possibility would be to insert repeated sequences flanking the restriction site into the plasmid and to biotinylate the linearized plasmid. After transport to the oocyte nucleus, such plasmids could readily circularize through homologous recombination. In the present study, developing a technique to produce gene knockout and knock-in, we exclusively used sigma constructs because we wanted to know whether end-biotinylated molecules bound to shuttle protein still recombine.

The DNA was transported to virtually all oocytes within an ovary. Thus, a single intravascular injection will produce several thousands of embryos carrying the donor DNA, which is an important condition for obtaining gene recombination. Indeed, the rate of spontaneous homologous recombination between a donor and an intact target gene is extremely low (around 10^−5^). Unless the transgene carries an easily detectable marker, it will be difficult to screen such a large number of offspring to find a few transgenic embryos.

Since recombination frequency is greatly enhanced by nicking the target gene, it would be useful to also transport the nicking agents into oocytes by means of the shuttle protein. To this end, one might attach the oocyte ligand of vitellogenin, RIIKSTDF (Li et al., 2003), to a freely accessible site of the ZFN or TALEN CRISPr/Cas proteins. Such modified proteins should home the oocyte nucleus along the vitellogenin pathway as does the shuttle protein. In the case of CRISPr/Cas, a technically attractive alternative would be to biotinylate the end of crRNA or tracrRNA protruding from the protein complex. Biotinylated CRISPr/Cas should then be carried to the oocyte by the OS6 shuttle protein. The agent nicking the target gene and the donor DNA could be mixed and applied in a single injection and the components necessary for efficient homologous recombination would reach the oocyte nucleus simultaneously.

At present, the oocyte shuttle approach is limited to vitellogenic species but might eventually be applied to mammalian species once specific ligands of proteins imported during oogenesis from the maternal blood are known. Females injected during pregnancy should then give birth to offspring carrying the donor DNA in their oocytes.

## MATERIALS AND METHODS

### Animal experimentation

The experiments respected Swiss and NIH guidelines and were under permit by the veterinary office of the State of Geneva.

### Oocyte shuttle protein

Plasmids were produced by standard techniques using enzymes from New England Biolabs, Bioconcept, Allschwil, Switzerland, and oligonucleotides were purchased from Microsynth, Balgach, Switzerland. To construct the plasmid coding for OS4, a synthetic oligonucleotide corresponding to the vitellogenin ligand, RIIKSTDF (Li et al., 2003), a 118 bp *Bsm*I/*Bbs*I fragment containing the nuclear localization signal and adjacent casein kinase phosphorylation site of the SV40 large tumor antigen, isolated from the *Hind*III plasmid 4002-5171, and the segment coding for red fluorescence from plasmid pDS Red1-N1, without stop codon or polyadenylation site, as well as the gene coding for a minimal sized core streptavidin from plasmid pTSA13 (Sano et al., 1995) were joined, if necessary by inserting linkers with appropriate restriction sites. The resulting OS4 gene (Fig. 1A) was cloned into pIVEX2.3 in front of the His tag. DNA. Amino acid sequences of OS4 are given in Fig. S1.

To visualize nuclear localization of the shuttle vector, the OS4-coding segment was placed under control of the CMV promoter of pDSRed N1, in place of the original coding sequences. The resulting CMV-OS4 plasmid was used for oocyte injection.

Because of low solubility of the OS4 protein, the variant OS6 was produced, (Fig. 1B; Fig. S2), in which DsRed was replaced by a linker. Both proteins were synthesized by custom baculovirus expression, custom service, AMS Biotechnology, Bioggio, Switzerland.

### Donor constructs

Plasmid Reco-σ was described in detail elsewhere (Hagmann et al., 1996) and is sketched in Fig. 1C. The plasmid was cut with *Bgl*II, and the 5′ end filled in by Klenow reaction with ^14biotin^dUTP and the other three dNTPs, followed by cutting with *Sac*I, eliminating the 1.1 kb spacer segment with the other biotinylated end.

To construct the EGFP-σ donor plasmid (Fig. 1D), the 3935 bp *Pci*I/*Bcg*I fragment, the 1099 bp *Pci*I/*Gsu*I fragment of pEGFP-C1, and a 46 bp spacer were joined by triple ligation. The repeated EGFP region in this plasmid is 306 bp long. The spacer carries stop codons and, among others, the unique restriction sites *Ac*cI and *Apa*I used for linearization of the donor and its biotinylation, filling in the 5′-protruding end of the *Acc*I site with dATP and ^14biotin^dUTP.

### Oocyte injection

*Xenopus laevis* females (2-4 years old) were from the Geneva *Xenopus* stock center, Station de Zoologie expérimentale, University of Geneva. Isolation of oocytes by collagenase treatment, centrifugation to visualize the position of the nucleus, buffers and injection procedure are described in detail elsewhere (Hagmann et al., 1996). To study cellular localization of the shuttle protein, pCMV-OS4 or pEGFP-C1 was injected into the nucleus of *albino* oocytes. Two days later, red or green fluorescence was visualized on a Zeiss Axiophot fluorescence microscope (Carl Zeiss AG, Feldbach, Switzerland).

To analyze nuclear translocation of donor DNA bound to the shuttle protein, 10 nl of injection buffer containing 0.5 ng of untreated or biotinylated Reco-σ with or without OS4 (0.05 ng of total protein preparation) were injected into the oocyte nucleus or cytoplasm. Note that the poorly soluble and dilute OS4 protein produced only faint band-shifts and the proportion of actively binding OS protein in our preparation could not be properly titrated. We thus used a sevenfold molar excess of protein over DNA, calculated by their respective molecular weights. After 18 h, batches of 20 oocytes were lysed, treated with proteinase K, and DNA purified on DNeasy columns (Qiagen, Hombrechtikon, Switzerland). Aliquots of the eluate were used to transfect *Escherichia* *coli* by electroporation and the bacteria were plated onto agarose plates containing 50 µg/ml ampicillin. The resistant colonies were then replica-printed on tetracycline plates (40 µg/ml) and Tet^R^ colonies were counted. Typically, positive points showed 20-100 colonies per oocyte depending on the experimental series.

### Intravascular injection into females

OS6 protein was used to titrate the actively binding shuttle protein by gel retardation experiments. A 30 bp oligonucleotide with ^7 biotin^dCTP on one 5′ end and fluorescent ^Cy5^dGTP on the other was used as probe. The bands were analyzed on 2% agarose gels and seized on a fluorescence scanner (Ettan Imager D10015 63-0056-42GE, Healthcare Life Sciences, Geneva, Switzerland).

Accordingly, for intravascular injection of females, 500 ng of biotinylated donor DNA and 70 ng of OS6 protein were incubated for 15 min at room temperature in 50 µl of 70% physiological salt solution (PBS). At this molar ratio, corresponding to the twentyfold excess yielding complete band-shift, the shuttle should bind all donor molecules (Reco-σ, 5300 bp; EGFP-σ, 5040 bp; OS6, 21.7 kDa). Since PBS was strongly diluted out in the final injection mix, it was not necessary to dialyze before use.

For intravascular injection, the females were primed with 50 IUof HCG and, 4 h later, anaesthetized (0.2% MS222). 50 µl of the DNA-protein mix were injected into their toe vein using a 29G BD micro-fine insulin syringe (Becton Dickinson, Allschwil, Switzerland). Ten minutes after injection, spawning was induced by 250IU of HCG. The eggs were discarded. 18 h after injection the females were euthanized and their oocytes isolated and probed for the presence of recombined Reco-σ molecules. Females having received EGFP-σ were kept for two weeks to allow for sufficient expression and maturation of green fluorescence. Ovaries or isolated oocytes were photographed under a Zeiss Axiophot microscope with equal exposure and identical image treatment in parallel.
